# Supplementary material for: Comprehensive generation, visualization, and reporting of quality control metrics for single-cell RNA sequencing data
Source: Nat Commun. 2022 Mar 30;13:1688. doi: 10.1038/s41467-022-29212-9 (PMC8967915; doi:10.1038/s41467-022-29212-9)
Supplement: Supplementary file 1 — Supplementary Information [file 41467_2022_29212_MOESM1_ESM.pdf]

| Preprocessing tools | Commandline | R console | Input folder layout |
|---|---|---|---|
| CellRanger | Rscript SCTK_runQC.R \ <br> -P **CellRangerV3** \ <br> -b **BasePath** \ <br> -o OutDirectory \ <br> -s **sample** ... <br><br> Rscript SCTK_runQC.R \ <br> -P **CellRangerV3** \ <br> -C **cellPath** \ <br> -R **rawPath** \ <br> -o OutDirectory ... | dropletSCE <- importCellRangerV3(cellRangerDirs = BasePath, sampleDirs = sample, sampleNames = sample, dataType = "raw", ...) <br><br> cellSCE <- importCellRangerV3(cellRangerDirs = BasePath , sampleDirs = sample, sampleNames = sample, ... dataType = "filtered") <br><br> dropletSCE <- importCellRangerV3Sample(dataDir = rawPath, ... ) <br> cellSCE <- importCellRangerV3Sample(dataDir = cellPath, ... ) | **BasePath** <br> [BasePath]/[sample]/outs/filtered_feature_bc_matrix/ <br> [BasePath]/[sample]/outs/raw_feature_bc_matrix/ <br><br> **rawPath** <br> [rawPath]/barcodes.tsv.gz <br> [rawPath]/features.tsv.gz <br> [rawPath]/matrix.mtx.gz <br><br> **cellPath** <br> [cellPath]/barcodes.tsv.gz <br> [cellPath]/features.tsv.gz <br> [cellPath]/matrix.mtx.gz |
| BUStools | Rscript SCTK_runQC.R \ <br> -P **BUStools** \ <br> -b **BasePath** \ <br> -o OutDirectory ... | dropletSCE <- importBUStools(BUStoolsDirs = BasePath, ...) <br> dropletSCE <- runDropletQC(inSCE = dropletSCE) <br> cellSCE <- dropletSCE[ , dropletSCE$dropletUtils_emptyDrops_fdr < 0.01] | **BasePath** <br> [BasePath]/genes.barcodes.txt <br> [BasePath]/genes.genes.txt <br> [BasePath]/genes.mtx |
| SEQC | Rscript SCTK_runQC.R \ <br> -P **SEQC** \ <br> -b **BasePath** \ <br> -o OutDirectory \ <br> -s **sample** ... | dropletSCE <- importSEQC(BUStoolsDirs = BasePath, prefix= sample, ...) <br> dropletSCE <- runDropletQC(inSCE = dropletSCE) <br> cellSCE <- dropletSCE[ , dropletSCE$dropletUtils_emptyDrops_fdr < 0.01] | **BasePath** <br> [BasePath]/[sample]_sparse_counts_barcodes.csv <br> [BasePath]/[sample]_sparse_counts_genes.csv <br> [BasePath]/[sample]_sparse_molecule_counts.mtx |
| Optimus | Rscript SCTK_runQC.R \ <br> -P **Optimus** \ <br> -b **BasePath** \ <br> -o OutDirectory ... | dropletSCE <- importOptimus(OptimusDirs = BasePath, ...) <br><br> cellSCE <- dropletSCE[,which(dropletSCE$dropletUtils_emptyDrops_IsCell)] | **BasePath** <br> [BasePath]/call-MergeCountFiles/ <br> [BasePath]/call-MergeCellMetrics/ <br> [BasePath]/call-MergeGeneMetric <br> [BasePath]/call-RunEmptyDrops/ |
| STARSolo | Rscript SCTK_runQC.R \ <br> -P **STARSolo** \ <br> -b **BasePath** \ <br> -o OutDirectory ... | dropletSCE <- importSTARsolo(STARsoloDirs = BasePath, STARsoloOuts = "Gene/raw", ...) <br><br> cellSCE <- importSTARsolo(STARsoloDirs = BasePath, STARsoloOuts = "Gene/filtered", ...) | **BasePath** <br> [BasePath]/Gene/raw/ <br> [BasePath]/Gene/filtered/ |
| DropEst | Rscript SCTK_runQC.R \ <br> -P **DropEst** \ <br> -b **BasePath** \ <br> -o OutDirectory ... | dropletSCE <- importDropEst(sampleDirs = BasePath, dataType="raw", ...) <br><br> cellSCE <- importDropEst(sampleDirs = BasePath, dataType="filtered", ...) | **BasePath** <br> [BasePath]/cell.counts.rds |
| CountMatrix | Rscript SCTK_runQC.R \ <br> -P **CountMatrix** \ <br> -c **cellFile** \ <br> -r **rawFile** \ <br> -o OutDirectory ... | dropletMM <- data.table::fread(dropletFile) <br> dropletSCE <- constructSCE(data = dropletMM, samplename = samplename) <br><br> cellMM <- data.table::fread(cellFile) <br> cellSCE <- constructSCE(data = cellMM, samplename = samplename) | **rawFile** <br> path/to/dropletMatrix.mtx <br><br> **cellFile** <br> path/to/cellMatrix.mtx |
| SCE | Rscript SCTK_runQC.R \ <br> -P **SceRDS** \ <br> -c **cellFile** \ <br> -r **rawFile** \ <br> -o OutDirectory ... | dropletSCE <- readRDS(rawFile) <br> cellSCE <- readRDS(cellFile) | **rawFile** <br> path/to/dropletSCE.rds <br><br> **cellFile** <br> path/to/cellSCE.rds |
| AnnData | Rscript SCTK_runQC.R \ <br> -P **AnnData** \ <br> -C **cellPath** \ <br> -R **rawPath** \ <br> -o OutDirectory \ <br> -s **sample** ... | dropletSCE <- importAnnData(sampleDirs = RawFile, sampleNames= "sampleRaw", ...) <br><br> cellSCE <- importAnnData(sampleDirs = CellFile, sampleNames= "sampleCell", ...) | **rawPath** <br> [rawPath]/sample.h5ad <br><br> **cellPath** <br> [cellPath]/sample.h5ad |
| Alevin | Rscript SCTK_runQC.R \ <br> -P **Alevin** \ <br> -b **BasePath** \ <br> -o OutDirectory \ <br> -s **sample** ... | cellSCE <- importAlevin(alevinDir = BasePath, sampleNames= "sample", ...) | **BasePath** <br> [BasePath]/alevin/quants_mat.gz <br> [BasePath]/alevin/quants_mat_cols.txt <br> [BasePath]/alevin/quants_mat_rows.txt <br> [BasePath]/alevin/reatureDump.txt |

**Supplementary Table 1. Import strategies of the SCTK-QC pipeline used to import data.** The last column demonstrates folder structure that is recognized by SCTK-QC pipeline for the dataset generated by each preprocessing tool. The first column shows the command-line implementation of the pipeline. The second column shows the script used to run the pipeline in the R console.

| | DoubletFinder removed | | DoubletFinder included | |
|---|---|---|---|---|
| | PBMC 34K | PBMC 68K | PBMC 34K | PBMC 68K |
| **Counts in dataset** | 33148 | 68579 | 33148 | 68579 |
| **Maximum memory necessary (GB)** | 6.0 | 7.0 | 18.5 | 33.5 |
| **SCE object size (MB)** | 660 | 950 | 660 | 950 |
| **System time (seconds)** | 2350 | 3960 | 4200 | 6820 |

**Supplemental Table 2. Benchmarking of SCTK Shiny User Interface.** The 34k and 68k PBMC datasets taken from the R/Bioconductor package TENxPBMC were analyzed with the Shiny UI for benchmarking of the QC functionality. Datasets were split into five batches of equal size, labeled as Samples 1-5 to simulate data from several samples comprising the 10X CellRanger output. For each dataset, QC algorithms *addPerCellQC*, *scrublet*, *scDblFinder*, *cxds*, *bcds*, *cxds_bcds_hybrid, and decontX* were applied. Two benchmarking runs were conducted, with one applying *DoubletFinder* and another not applying *DoubletFinder*.
