# Supplementary material for: Comprehensive generation, visualization, and reporting of quality control metrics for single-cell RNA sequencing data
Source: Nat Commun. 2022 Mar 30;13:1688. doi: 10.1038/s41467-022-29212-9 (PMC8967915; doi:10.1038/s41467-022-29212-9)
Supplement: Supplementary file 2 — Reporting Summary [file 41467_2022_29212_MOESM2_ESM.pdf]

nature research

Corresponding author(s): Campbell, Joshua D.

Last updated by author(s): Feb 17, 2022

Reporting Summary

Nature Research wishes to improve the reproducibility of the work that we publish. This form provides structure for consistency and transparency in reporting. For further information on Nature Research policies, see our Editorial Policies and the Editorial Policy Checklist.

Statistics

For all statistical analyses, confirm that the following items are present in the figure legend, table legend, main text, or Methods section.

n/a	Confirmed	
☐	☒	The exact sample size (n) for each experimental group/condition, given as a discrete number and unit of measurement
☐	☒	A statement on whether measurements were taken from distinct samples or whether the same sample was measured repeatedly
☐	☒	The statistical test(s) used AND whether they are one- or two-sided<br>*Only common tests should be described solely by name; describe more complex techniques in the Methods section.*
☒	☐	A description of all covariates tested
☐	☒	A description of any assumptions or corrections, such as tests of normality and adjustment for multiple comparisons
☐	☒	A full description of the statistical parameters including central tendency (e.g. means) or other basic estimates (e.g. regression coefficient) AND variation (e.g. standard deviation) or associated estimates of uncertainty (e.g. confidence intervals)
☐	☒	For null hypothesis testing, the test statistic (e.g. F, t, r) with confidence intervals, effect sizes, degrees of freedom and P value noted<br>*Give P values as exact values whenever suitable.*
☒	☐	For Bayesian analysis, information on the choice of priors and Markov chain Monte Carlo settings
☒	☐	For hierarchical and complex designs, identification of the appropriate level for tests and full reporting of outcomes
☒	☐	Estimates of effect sizes (e.g. Cohen's d, Pearson's r), indicating how they were calculated

Our web collection on statistics for biologists contains articles on many of the points above.

Software and code

Policy information about availability of computer code

Data collection	10X Genomics CellRanger v3.1.0
Data analysis	scrublet v0.2.2, scater v1.4.0, DoubletFinder v2.0.3, scDblFinder v1.8.0, scds v1.10.0, celda v1.10.0, DropletUtils v1.14.1

For manuscripts utilizing custom algorithms or software that are central to the research but not yet described in published literature, software must be made available to editors and reviewers. We strongly encourage code deposition in a community repository (e.g. GitHub). See the Nature Research guidelines for submitting code & software for further information.

Data

Policy information about availability of data

All manuscripts must include a data availability statement. This statement should provide the following information, where applicable:
- Accession codes, unique identifiers, or web links for publicly available datasets
- A list of figures that have associated raw data
- A description of any restrictions on data availability

Data was obtained from the following websites:
https://www.10xgenomics.com/resources/datasets/1-k-pbm-cs-from-a-healthy-donor-v-2-chemistry-3-standard-3-0-0 (10X V2)
https://www.10xgenomics.com/resources/datasets/1-k-pbm-cs-from-a-healthy-donor-v-3-chemistry-3-standard-3-0-0 (10X V3)
https://singlecell.broadinstitute.org/single_cell/study/SCP424/single-cell-comparison-pbmc-data (SMART-Seq2)

Field-specific reporting

Please select the one below that is the best fit for your research. If you are not sure, read the appropriate sections before making your selection.

☒ Life sciences ☐ Behavioural & social sciences ☐ Ecological, evolutionary & environmental sciences

For a reference copy of the document with all sections, see nature.com/documents/nr-reporting-summary-flat.pdf

Life sciences study design

All studies must disclose on these points even when the disclosure is negative.

Sample size	We utilized 4 publicly available single cell RNA-seq PBMC datasets containing over 1000 cells each to demonstrate the utility of our software.
Data exclusions	No data was excluded.
Replication	We demonstrated the ability of our software to work on 2 PBMC datasets profiled with 2 different 10X protocols. Furthermore, each of these datasets was processed using 2 different reference genomes. Finally, we showed the ability of our software to work on another scRNA-seq generated by the SMART-seq protocol.
Randomization	Randomization was not necessary for this study as the goal was to show that the output of the software is robust on many different types of data.
Blinding	Blinding was not relevant to the study, as the goal of this study was the development of a quality control pipeline software, and no statistical analysis is reported outside.

Reporting for specific materials, systems and methods

We require information from authors about some types of materials, experimental systems and methods used in many studies. Here, indicate whether each material, system or method listed is relevant to your study. If you are not sure if a list item applies to your research, read the appropriate section before selecting a response.

Materials & experimental systems

n/a	Involved in the study
☒	☐ Antibodies
☒	☐ Eukaryotic cell lines
☒	☐ Palaeontology and archaeology
☒	☐ Animals and other organisms
☒	☐ Human research participants
☒	☐ Clinical data
☒	☐ Dual use research of concern

Methods

n/a	Involved in the study
☒	☐ ChIP-seq
☒	☐ Flow cytometry
☒	☐ MRI-based neuroimaging
